# Maternal mortality in Pakistan: Challenges, efforts, and recommendations

**DOI:** 10.1016/j.amsu.2022.104380

**Published:** 2022-08-18

**Authors:** Sean Kaisser Shaeen, Zoaib Habib Tharwani, Wajeeha Bilal, Zarmina Islam, Mohammad Yasir Essar

**Affiliations:** Faculty of Medicine, Dow University of Health Sciences, Karachi, Pakistan; Faculty of Medicine, Dow University of Health Sciences, Karachi, Pakistan; Faculty of Medicine, Dow University of Health Sciences, Karachi, Pakistan; Faculty of Medicine, Dow University of Health Sciences, Karachi, Pakistan; Kabul University of Medical Sciences, Kabul, Afghanistan

**Keywords:** Pakistan, Maternal mortality, Healthcare, Antenatal care, Postnatal care

## Abstract

Maternal mortality is a major concern in various countries across the globe and particularly Pakistan. Regardless of the fact that the maternal mortality rate is steadily decreasing over time, Pakistan still faulters below the progress necessary to reach its set goals. Challenges such as lack of skilled staffing, the facilities to house them, lack of education as well as social constraints are proving to be significant obstacles for the healthcare system of Pakistan and its attempts to reduce the maternal mortality rate. Furthermore, the rural environments of the country, poverty constraints, and general ignorance continue to exacerbate the factors previously listed. In order to address these issues, the government needs to work alongside international organizations to acquire funding to build new facilities, particularly in rural areas, train skilled staff, educate on the benefits of antenatal and delivery care, and provide additional funding to subsidize the care itself for those in need. This with further research done in order to assess the effectiveness of various programs and the progress over time will make an impact on reducing the maternal mortality rate in Pakistan.

## 1 Introduction

Over the years maternal mortality ratio (MMR) worldwide has decreased by 38% from 2000 to 2017 [1]. Maternal mortality, as defined by the World Health Organization (WHO), is death during pregnancy or death within 42 days after termination of pregnancy. The cause of death is unrelated to the duration and site of pregnancy, and may be caused by any condition related to or worsened by the pregnancy or its management, but is not related to unintentional or incidental causes [1]. The major complications which cause maternal mortality include: cardiomyopathies, pulmonary or other embolisms, hemorrhage, infection or sepsis, pre-eclampsia & eclampsia, complications from delivery, and unsafe abortion [1,2].

Even though MMR has shown a significant decrease over the past years, the rates still remain high in low- & middle-income countries such as Afghanistan (638/100,000 livebirths), India (145/100,000 livebirths), Bangladesh (173/100,000 livebirths), and Yemen (164/100,000 livebirths) [[3], [4], [5], [6]]. Pakistan, being one of the low-income countries, also reported a high MMR of 186 deaths per 100,000 livebirths in 2019, a 32% increase from 2017 (140/100,000 livebirths) [7,8]. Most affected were specifically the rural areas, with an MMR of 199 per 100,000 livebirths, as compared to the urban areas with an MMR of 158 per 100,000 livebirths [8].

One of the major factors that affected the MMR was the COVID-19 pandemic. The pandemic has affected nearly 1.5 million people in Pakistan to date, with more than 30,000 deaths being reported [9]. Due to COVID-19, Pakistan's healthcare system suffered from shortage of beds, lack of medicine and oxygen cylinders, as well as attacks on healthcare workers [10]. Moreover, due to the lockdowns, a lot of businesses and industries were affected which led to a rise in poverty rates, thus making better healthcare inaccessible to many, furthering the rise in the country's MMR [11].

Although MMR in Pakistan has shown a drastic improvement from the year 2000–2017, a rise recorded in 2019 is of great concern [7,8]. Moreover, after 2020 (during COVID-19) various factors have affected the healthcare system in general, also hindering the control of MMR in Pakistan. Therefore, this article aims to highlight those factors and establish a correlation between them and MMR, and also aims to provide recommendations for further decreasing the MMR in Pakistan.

## 2 Challenges

Pakistan has one of the highest maternal mortality rates in South Asia [12]. Despite being a signatory of Agenda 2030, the country still lags considerably behind when it comes to achieving Sustainable Development Goals (SDGs) [12]. Approximately 20% of the deaths that occur among women of childbearing age are related to maternal complications [13]. The vast majority of these cases are due to postpartum hemorrhage. This can be extremely problematic if hospitals do not have enough blood to transfuse to replace the lost blood [13]. Maternal mortality is also high due to puerperal sepsis and eclampsia. In sepsis, also known as blood poisoning, infections during pregnancy trigger the body's inflammatory response to infection and can only be cured with prompt treatment with antibiotics [13]. Often followed by coma, eclampsia, or high blood pressure causing convulsions, is the third leading cause of maternal death in Pakistan [13]. Additionally, delays in medical care during obstetric complications is also a factor that leads to maternal deaths. One such example of delay comes in the form of household constraints or rather it is the ignorance of families and traditional midwives that delay the decision to seek medical care [14]. A second form of delay occurs when women are unable to get to hospitals due to a lack of telephones and ambulance services [14]. Lastly, delay at the hospital setting itself can be caused mainly by the lack of trained staff, supplies and equipment, as well as poorly organized emergency services [14].

Pakistan Maternal Mortality Survey 2019, conducted by the National Institute of Population Studies and funded by the United States Agency for International Development (USAID), reveals considerable demographic differences between rural and urban areas of Pakistan [12]. The MMR is nearly 26% higher in rural areas as compared to urban areas due to a major difference in health care services provided to people living in urban areas as compared to those living in distant regions [12]. A recent survey revealed that 62% of rural women of reproductive age are uneducated, which is significantly higher than the percentage of uneducated urban women [12]. This is particularly pertinent as women who are educated are more likely to seek prenatal care. In Pakistan, approximately 96% of women with education received prenatal care from a doctor, compared to 50% of women without education 13i. Aside from education, another contributing factor to the MMR is cost as evident by the fact that a third of pregnant women in Pakistan do not seek prenatal care specifically because it is deemed unnecessary or more importantly, too expensive. This warrants concern as prenatal care can reduce the rates of complications and maternal mortality [13]. Finally, the lack of doctors, nurses, and beds in government hospitals also contributes to the high maternal mortality rate in the country. Most regular staff members are postgraduate trainees incapable of dealing with pregnancy-related complications. This is presumably due to inadequate experience and exposure [13].

Rural women lack access to essential healthcare facilities. Many of them die while traveling to far-off hospitals located in cities. As Pakistan currently spends less than 1% of its GDP on health care [13], more funds must be devoted to improving hospital care and making hospitals more accessible. The rate of maternal mortality is consequently higher in rural areas than urban areas—23% rather than 14% [13]. Home births are extremely common in rural areas. A total of 74% of women in rural areas give birth at home, compared to 43% of women in urban areas [13]. Additionally, poverty, rampant in rural areas, is a factor in the prevalence of women seeking healthcare facilities during, before, or after pregnancy, mainly because they lack access to incomes, clothing, education, proper sanitation, and human rights [14]. It is therefore imperative that political stability be ensured and long-term policies are made to develop healthcare infrastructure in these unprivileged areas.

Many Pakistani women, especially those living in rural areas, are frequently vulnerable to old customs and outdated cultural practices that sometimes lead to their deaths. Females who are illiterate and live in rural areas of Pakistan are more likely to get married early because of child marriage before they reach the legal age of marriage as per law which is 18 for men and 16 for women, although women are sometimes allowed to marry at the age of fourteen [15]. These women are unlikely to take advantage of maternal health services provided by trained medical staff and therefore deliver their babies at home, which is associated with an increased risk factor contributing to maternal morbidity and mortality [12]. A number of studies have shown that family planning reduces the number of births, and thus, the number of times a woman is exposed to the risk of mortality. There has not been much increase in the use of contraceptives in recent years, which could also explain Pakistan's high maternal mortality rate. Its usage has been steady, and only about 30% of married women of childbearing age use contraceptives [13]. Women who are educated are more likely to use contraceptives, but many women in Pakistan are uneducated, explaining why the fertility rate in impoverished households is typically higher. They undergo unsafe or traditional birth practices performed by unskilled persons more frequently, and they exhibit less evidence of following a family planning regimen [14].

## 3 Efforts and recommendations

Pakistan's government and its people, as well as other international organizations, have made various attempts to reduce the maternal mortality rate within its country. Pakistan's federal and provincial governments have set the goal of reducing the maternal mortality rate of 70 deaths per 1000,000 live births by the year 2030 [16]. Some changes that have been made include the increase in Antenatal care and delivery care coverage for women in need as the number of skilled antenatal care providers have increased from 26% in 1991 to 91% as of 2019 [16]. The number of deliveries held in healthcare facilities have also significantly increased from 14% in to 71% as of late [16]. Furthermore, the government has created the Pakistan Maternal Mortality Survey (PMMS) with the goal of providing vital data to continually assess the progress being made towards their end goal in 2030. Within this survey, they made a well-constructed definition on maternal mortality, collected data to check on the progress of the goal of reducing maternal mortality, find the common causes of it, and find the differences between the rates in rural versus urban areas.

Asides from the government's attempts to reduce the rate of maternal mortality, local organizations have also made strides with the same goal. For example, the National Committee for Maternal and Neonatal Health (NCMNH) has multiple roles it plays to reduce the maternal mortality ratio as well as improve maternal health overall [17]. The NCMNH does this through various means such as providing technical assistance to the government, helping with policy making, planning, implanting and even monitoring programs that are ultimately directed at the improvement of maternal health and the reduction of the maternal mortality rate [17]. Additionally, The United Nations Population Fund (UNFPA) does a lot for women and children across the board for those in Pakistan. Some ways in which they help reduce maternal mortality generally include advocating for health and education, reducing gender gap inequality, and changing social norms. More direct approaches include the increase in the provision of skilled midwifery services specifically on low-income groups [18].

Recommendations for reducing the maternal mortality rate in Pakistan are numerous. They start with first increasing the budget and funding in healthcare while prioritizing women's reproductive health particularly in rural areas. The finances can be used to increase training programs that work to provide skilled antenatal and delivery care as well as midwifes in general. It can also be used to increase the number of healthcare facilities themselves as citizens living in rural areas tend to have less facilities available and have to travel greater distances to reach said facilities compared to those living in an urban environment. This is essential as Pakistan is an agricultural country with 64% of its people living in rural areas [19]. Moreover 62% of rural women are uneducated compared to only 34% of their urban counterparts. This is compounded by the fact that 25% of the population lives below the poverty line, with a majority of which are living in rural areas [19]. Therefore, it is quintessential that governments and organizations focuses on providing funding, particularly in rural areas, to educate more qualified healthcare workers, produce more facilities to house women, educate the locals about the importance of antenatal, delivery, and postpartum care. Simultaneously, the government and other parties involved should provide separate funding to give access to those individuals in need or to at least subsidize said services who could otherwise not afford them. Additionally, aside from training healthcare professionals, the government and or healthcare professionals themselves can host training sessions for those interested that provide a basic understanding of natal care such that they can provide first aid and assist in the birthing process in case the need arrives. This would be especially helpful in rural areas as these individuals are more likely to find it hard to travel to distant healthcare facilities, are unable to afford it, or are in a sudden and unforeseen medical emergency scenario. Finally, it is important to understand that the lack of healthcare facilities and education in general invites various complications. A significant one being that such an environment leads to infections such as Covid-19 being spread quicker and being harder to manage as well as vaccination distribution being a particularly arduous task. This can and has resulted in additional deaths particularly maternal deaths as its been reported by the United Nations that due to covid, an estimated 11,000 additional maternal deaths were expected in 2020 [20].

## 4 Conclusion

In conclusion, Pakistan has made performed well in reducing the maternal mortality rate throughout the years through various means, however, it is still one of the few countries which have a comparatively high rate. Therefore, in order to continue reducing the rate, it is essential that the national and provincial government works with international organizations to thoroughly fund new facilities, educate to create more qualified healthcare workers and educate the masses in the benefits of specialized care and finally provide financial assistance to those who would otherwise be unable to afford said services.

## Ethical approval

N/A.

## Sources of funding

None.

## Author statement

Sean Kaisser Shaeen conceived the idea, came up with the design, wrote the abstract, efforts, recommendations, conclusion, and edited the revised draft and organized references; Zoaib Habib Tharwani wrote the introduction; Wajeeha Bilal wrote the challenges; Zarmina Islam gave essential comments and guidance; Mohammad Yasir Essar made the critical comments and revision. All authors revised and approved the final draft.

## Registration of research studies

1. Name of the registry: N/A
2. Unique Identifying number or registration ID: N/A
3. Hyperlink to your specific registration (must be publicly accessible and will be checked): N/A

## Guarantor

N/A.

## Consent

N/A.

## Provenance and peer review

Not commissioned, externally peer reviewed.

## Conflicts of interest

None declared.

## Abbreviations

MMR: Maternal Mortality Ratio.
USAID: United States Agency for International Development.
PMMS: Pakistan Maternal Mortality Survey.
NCMNH: The National Committee for Maternal and Neonatal Health.
UNFPA: The United Nations Population Fund.
